# New Evidence on Nutrition and Metabolic Influences on Pregnancy Outcomes: Insights from This Special Issue

**DOI:** 10.3390/nu17162628

**Published:** 2025-08-14

**Authors:** Jose Luis Bartha

**Affiliations:** 1 Department of Obstetrics and Gynecology, University Hospital La Paz, 28046 Madrid, Spain; josel.bartha@salud.madrid.org or jose.bartha@uam.es; 2 Faculty of Medicina, Universidad Autónoma of Madrid, 28046 Madrid, Spain

The complex relationship between maternal nutrition, metabolic status and pregnancy outcomes has long remained controversial in perinatal research. With increasing global concern about the rise in obesity, gestational diabetes mellitus (GDM) and fetal macrosomia, and the growing new data about how dietary and metabolic factors influence the development of placental disorders such as preeclampsia or intrauterine growth restriction (IUGR), interest in this topic is paramount. This Special Issue of *Nutrients*, “Nutritional and Metabolic Factors in Pregnancy Complications”, brings together eight original and varied investigations that may help to understand important aspects of this multifaceted interplay. As the Guest Editor, I present this Editorial not only to summarize the main findings of these studies, but also to place them within the context of maternal and fetal medicine.

## 1. Cardiovascular Risks and Lifestyle

The ECLIPSES Study, led by Motevalizadeh et al. [contribution 1], evaluated the associations between lifestyle, anthropometric and socioeconomic factors, and clustered cardiometabolic risk among pregnant women. Their findings confirmed that pregestational overweight and obesity, sedentary lifestyle, and low socioeconomic status are significantly associated with increased cardiovascular risk during pregnancy. Notably, this study highlights the accumulative impact of modifiable behaviors and socioeconomic determinants, supporting the need for public health policies that encourage early lifestyle interventions, particularly in vulnerable populations.

Along the same lines, Yuste et al. [contribution 2] examined dietary patterns and components of a Mediterranean diet to provide further evidence of the protective role of a well-balanced diet full of rich nutrients on the prevention of GDM. Importantly, high adherence to this pattern was associated with improved glycemic profiles. The authors emphasized how nutritional counseling before and during pregnancy can be an effective strategy for metabolic disease prevention during and probably after pregnancy.

## 2. Metabolic Role in Placental Disorders

A deeper insight into the role of metabolic factors in the pathophysiological mechanisms of placental-related disorders is presented by Abascal-Saiz et al. [contribution 3], who evaluated placental fatty acid oxidation (FAO) gene expression in pregnancies complicated by preeclampsia and IUGR, distinguishing between cases with either early or late presentation that seemed to have different origins. The authors found a marked downregulation of key FAO genes, particularly in severe early-onset forms, suggesting impaired mitochondrial function and lipid metabolism alterations in the placenta. These results, together with some others by the same group of researchers (and others), add to the mechanisms underlying these conditions, supporting the important role of nutrients and placental metabolism and how impaired placental metabolism and dysfunction may co-evolve, laying the groundwork for future therapeutic targeting, including the implementation of a Mediterranean diet and potentially drugs for lipid metabolism alterations.

In parallel, González González et al. [contribution 4] contributed with an innovative methodological tool by validating customized fetal growth charts that incorporate maternal height, weight, ethnicity and parity. This individualized approach allows for more accurate differentiation between constitutionally small and growth-restricted fetuses, thus refining the assessment of IUGR risk when combining maternal and fetal nutritional characteristics. The clinical utility of these tools lies in improving perinatal outcomes by optimizing monitoring and intervention strategies.

## 3. Micronutrients and Thyroid Function

Iodine and selenium, trace elements essential for thyroid function and fetal neurodevelopment, emerged as central factors in several studies. Shenhav et al. [contribution 5] evaluated the prevalence of iodine deficiency and its impact on maternal thyroid hormone levels and function. Their finding revealed that even mild iodine deficiency was associated with altered thyroid profiles, emphasizing the vulnerability of pregnant women to micronutrient insufficiencies and the importance of dietary supplementation or diet fortification.

Ovadia et al. [contribution 6], on the same topic, evaluated the interactions between iodine intake, carbohydrate consumption, and neonatal outcomes. They found that in iodine deficiency, maternal dysglycemia was more strongly associated with fetal overgrowth. This synergistic effect underscores the complexity of metabolic programming and suggests that micronutrient status may modulate the impact of maternal glycemic control and fetal growth.

Moreover, Hao et al. [contribution 7] conducted a detailed trace elements analysis of placental tissues from preeclamptic pregnancies. Their data revealed significantly reduced levels of magnesium, zinc, and selenium, nutrients known to participate in vascular and antioxidative functions. These findings suggest that placental insufficiency in preeclampsia may be exacerbated by local nutrient deficiencies, pointing toward either potential adjunctive therapies or predictive biomarkers.

## 4. Metabolic Impact of COVID-19 and Infectious Burden

A new contribution from González González takes a timely look at the impact of recent COVID-19 infection on metabolic profiles during pregnancy [contribution 8]. By analyzing fasting glucose levels in post-COVID-19 pregnancies, the study found elevated glycemic markers, even among women without prior metabolic diseases. This raises concerns about the persistent metabolic effects of viral infections and their contribution to GDM risk. The findings are particularly relevant as we continue to evaluate the long-term implications of SARS-CoV-2 exposure during gestation.

## 5. Summary and Conclusions

Taken together, these eight articles in this Special Issue offer a comprehensive view of how nutritional and metabolic factors may contribute to pregnancy complications, encompassing not only metabolic but also placental-related disorders. They evaluate molecular insights, epidemiological patterns, clinical tools, and public health policies. Despite their diversity, they have many similarities that provide a rational for this unifying issue. These include the importance of early intervention, both preconceptionally and in early pregnancy, the synergistic effects of micronutrients, metabolism, and endocrine function that highlight the need for integrative nutritional strategies, novel diagnostic tools such as personalized fetal charts and molecular markers of placental health that show promise in risk stratification, and the evolving impact of infectious diseases like COVID-19 on maternal metabolism, which needs ongoing surveillance and new care protocols.

In summary, this Special Issue underscores the essential role of nutrition and metabolism in determining maternal, fetal, and neonatal health. As research continues to uncover the intricate biological, behavioral, and social contributors to pregnancy complications, it becomes clear that a multidisciplinary approach is required. Nutritional interventions, metabolic monitoring, and personalized care models should become integral components of maternal healthcare. We hope that the contributions collected here will not only update clinical practice, but also inspire further research to improve the health and well-being of pregnant women and children.

